# Fabrication of Nanoyttria by Method of Solution Combustion Synthesis

**DOI:** 10.3390/nano10050831

**Published:** 2020-04-27

**Authors:** Magdalena Gizowska, Milena Piątek, Krzysztof Perkowski, Gustaw Konopka, Irena Witosławska

**Affiliations:** 1 Department of Ceramics and Composites, Division of Ceramic and Concrete in Warsaw, Łukasiewicz Research Network—Institute of Ceramics and Building Materials, 9 Postępu Street, 02-676 Warsaw, Poland; k.perkowski@icimb.pl (K.P.); i.witoslawska@icimb.pl (I.W.); 2 Research Laboratory, Division of Ceramic and Concrete in Warsaw, Łukasiewicz Research Network—Institute of Ceramics and Building Materials, 4 Kupiecka Street, 03-042 Warsaw, Poland; m.piatek@icimb.pl (M.P.); g.konopka@icimb.pl (G.K.)

**Keywords:** DIL, DTA, DTG, FT-IR, SCS, yttria nanopowder

## Abstract

In the work the research on properties of an yttria nanopowder obtained by solution combustion synthesis (SCS) in terms of its application in ceramic technology is presented. In order to characterize the SCS reaction the decomposition of yttrium nitrate, glycine and their solution was investigated using differential thermal analysis coupled with FT-IR spectrometry of the gases emitted during the measurements. The product obtained in the SCS process was characterized in terms of its microstructure, particle size distribution and BET specific surface. Although the obtained powders showed nanoscaled structures, only after calcination at a temperature of 1100 °C nanosized particles were revealed. The calcined powder occurred in an agglomerated state (cumulants mean Z_ave_ = 1.3 µm). After milling particle size was successfully decreased to Z_ave_ = 0.28 µm. The deagglomerated powder was isostatically densified and tested for sintering ability. The obtained nanopowder showed very high sintering activity as the shrinkage onset was detected already at a temperature of about 1150 °C.

## 1. Introduction

A wide variety of applications of yttria is a driving force for the development of methods to fabricate pure powders. Doped or surface modified yttria nanoparticles find application in medicine and electronics [1,2,3,4].

Ceramic nanoparticles recently gained interest as an reinforcement of light weight alloys. Lately it was proven that the addition of 2.5 wt% ceramic nanoparticles caused an increase of the microhardness of Ti6Al4 V by 50%, manufactured by selective laser melting (SLM) [5]. Due to its high hardness and low reactivity with molten metals yttria can be an interesting material for metal matrix composites. Additionally, yttria has a relatively high thermal conductivity (8–12 W/m·K), which in case of the SLM technique can prove to be beneficial for the forming and densification of produced parts due to better heat transfer in the area of the laser’s operation.

Yttria is also widely used in ceramic technology. Dense yttria ceramics find application as refractive ceramics (e.g., coatings and crucibles for molten reactive metals), optic devices (i.e., infrared missile domes) [6,7]. For ceramic technology nanopowder is desired due to the possibility of reducing the sintering temperature [8].

Solution combustion synthesis (SCS), which is based on the high energy reaction between metal nitrates and a reducing agent, is a promising method for the fabrication of nanopowders. Unlike sol-gel or precipitation technique [9,10] it is less time-consuming and requires fewer technological steps, as the synthesis byproducts undergo thermal decomposition. The principles of the SCS reaction are also used in technical scale and in continuous technology [11,12]. Recently, the mixture of an oxidizer-reducing agent solution has been used for the production of a powder in aerosol flame synthesis and spray pyrolysis [13,14,15]. The self-propagating high-temperature reaction is used in order to provide finer structuration of the powders produced in these methods.

A particularly interesting application of the SCS principle is a technique used to fabricate thin films for electronic devices such as solar cells, where the surface is coated with the solution containing the oxidizer–“fuel” mixture which is subsequently heated to produce a uniform oxide film [16].

The SCS method is based on the high-temperature self-propagating red-ox reaction between a metal nitrate and a reducing agent (“fuel”), which leads to the fabrication of metal oxide or its precursor, according to the overall SCS formula [12], non-toxic gas products. In the role of fuel, the following substances can be used: carbohydrazide, urea, amino acids (e.g., glycine, L-alanine) organic acids and saccharides [17,18,19,20].

Based on previous research, glycine is the most promising fuel for SCS reaction fuel for the fabrication of yttria [21]. The general formula of the reaction of yttrium nitrate and glycine is as follows (1):

6 Y (NO_3_) _3_ + 10 NH_2_CH_2_COOH → 3 Y_2_O_3_ +20 CO_2_ + 25 H_2_O +14 N_2_ (1)

During the reaction vast amounts of gases are emitted which causes nanostructuration of the produced grains. Usually, the powders obtained by using this method show a high specific area and very complex particle morphology. Such microstructure is not beneficial for the fabrication of dense ceramics. Densification of nanopowders is a challenge in itself, as the smaller the particle, the more the particle-particle contact and the torque occurring between particles hinders the packing. Additionally, agglomerated particles need even higher pressures during compaction in order to destroy the inner structure of the particles. In the presented work we prove that with carefully designed technological steps the yttria powder obtained by using the SCS method has sintering ability and can be used for a ceramic application.

## 2. Materials and Methods

For the solution combustion synthesis of yttria yttrium nitrate hexahydrate (Sigma-Aldrich, St. Louis, MO, USA, purity 99.8%) and glycine (Sigma-Aldrich, purity ≥ 99%) was used. Glycine was used as the reducing agent and yttrium nitrate was both the precursor salt for yttria synthesis and the oxidizer in the redox reaction.

The solution combustion synthesis (SCS) was carried out in a quartz beaker. After the dissolution of the reagents in deionized water, the water was evaporated, and a gel was formed. The gel was then heated to the reaction initiation temperature. Once that temperature was reached, the high-temperature, self-propagating redox reaction took place [22]. The substrates were added in stoichiometric amounts, in each batch the aim was to obtain 5 g of yttria.

Thermogravimetric analysis was carried out in alumina crucibles using the thermal analyzer TG 449 F1 Jupiter (Netzsch Gerätebau GmbH, Selb, Germany). The gaseous products emitted were analyzed by FT-IR spectroscopy using a coupled FT-IR spectrometer (Tensor 27, Bruker, Billerica, MA, USA). The signals were identified based on the NIST database [23] and literature on glycine decomposition [24]. The curves of intensity of the characteristic absorbance wavenumber of a specific substance were subtracted and plotted as a function of temperature to investigate the reaction mechanism.

The powders’ microstructure was characterized utilizing scanning electron microscopy (Nova NanoSEM 200, FEI Company, Hillsboro, OR, USA).

Obtained powders were deagglomerated in an attritor mill (Netzsch MiniCer, 1000 rpm) using zirconia balls of diameter of 0.4 mm. The milled powders mixed with binder and plasticizer were cryogranulated.

The particle size distribution of the powders was measured in aqueous suspensions by technique of dynamic light scattering (DLS), using zeta potential analyzer Zetasizer Nano ZS (Malvern Instruments Ltd., Worcestershire, UK). The analysis results are presented in terms of Z average (Z_ave_) and polydispersity (Pd). These are supported by a median particle diameter (d_V50_). Z average (also called the cumulants mean or harmonic intensity averaged particle diameter) is a mean value from the intensity distribution, which is the primary result obtained from the measurement thus the most stable result. Polydispersity derives from the polydispersity index (calculated from the cumulants analysis) and is the width of the estimated Gaussian distribution.

The specific surface area was measured by use of the BET technique (Gemini VII, Micromeritics Instrument Corp., Norcross, GA, USA). Based on the results of the BET, the equivalent spherical-particle diameter (d_BET_) was calculated.

The measurement of linear changes of pressed granulates were conducted using a Netzsch high-temperature dilatometer (model Dil 402E) equipped with a graphite furnace. The measurement was carried out in a temperature range of RT to 1700 °C with a heating rate of 10 °C/min and the isothermal stage at the maximum temperature for a duration of 10 min.

Prior to measurement, calibration was carried out with a graphite standard of known properties and expansion. The measurement was carried out under the same conditions (temperature heating program, atmosphere, gas flow rate) to determine the signals related to the expansion of the dilatometer elements and to correct the results obtained during the proper measurement.

## 3. Results

**Figure 1:**
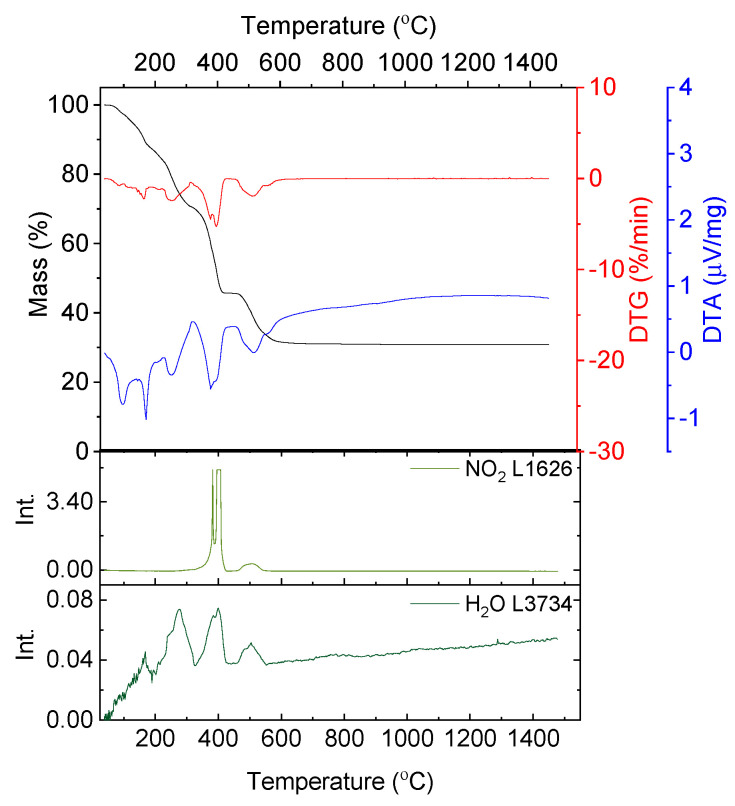
Thermal analysis results of the decomposition of yttrium nitrate hexahydrate in argon flow; absorbance intensities of traces for wavenumbers: 1626 and 3734 cm^−1^ corresponding with absorbance peaks of NO_2_ and H_2_O, respectively.

Figure 1 shows the results of thermal analysis for yttrium nitrate hexahydrate. In Figure 1 in the top graph the curves corresponding to mass loss, mass loss derivative and thermal effects of thermal decomposition of yttrium nitrate hexahydrate are presented. The graphs below represent the absorbance intensity trends of selected wavenumbers as a function of temperature.

In the gaseous products resulting from yttrium nitrate decomposition water and nitrogen dioxide are detected. The first endothermic effect detected on the DTA curve (Figure 1) corresponds with the melting of the salt. Minor weight loss is then observed (3.44%) related to the evaporation of adsorbed water and the small signal on the DTG curve with a minimum at a temperature of 87 °C. At a temperature of about 108 °C dehydration begins and is followed in two stages (Figure 1):

- 108–193 °C with a maximum of mass loss rate at a temperature of 166.1 °C and endothermic peak at 170.8 °C, Δm_108–193 °C_ = 9.48%,
- 193–327 °C with a maximum of mass loss rate at a temperature of 267.5 °C and endothermic peak at 273.9 °C, Δm_193–327 °C_ = 18.74%.

In the temperature range of 108–327 °C the total mass loss is 28.12% which is close to the theoretical value of the complete dehydration of the salt (28.20%), which is confirmed by FT-IR data since exclusively the signal of water is visible (Figure 1).

Further mass loss occurs in two steps and corresponds to the degradation of the nitrate. The first distinctive mass loss (Δm = 25.16%) occurs in the temperature range of 327–444 °C with a maximum mass loss rate at T = 397.7 °C and an endothermic peak at T = 398 °C (Figure 1). During the last decomposition stage, a mass loss of 13.32% occurs with a maximum mass loss rate at a temperature of 521.7 °C and an endothermic peak of 521.8 °C. Above the temperature of 641 °C the mass of the sample is stable. In both stages the signal indicating the presence of NO_2_ is visible. Yttrium nitrate primarily decomposes to yttria and nitrogen pentaoxide, which is unstable and converts to nitrogen dioxide and oxygen. The two observed steps are a consequence of partial decomposition and the forming of cyclic oxynitrates [25].

In the temperature range of 327–641 °C the total mass loss equals 38.48%, which is in good consistency with the theoretical value of the decomposition reaction stoichiometry (42.30%) (2).

(2) $$2\;Y({\mathrm{NO}}_{3}){\;}_{3}\;\overset{T}{\to }\;Y_{2}O_{3}+3\;N_{2}O_{5}$$

Total mass loss observed during the decomposition of yttrium nitrate hexahydrate is 69.68% and corresponds well to the theoretical value of mass loss (70.52%).

**Figure 2:**
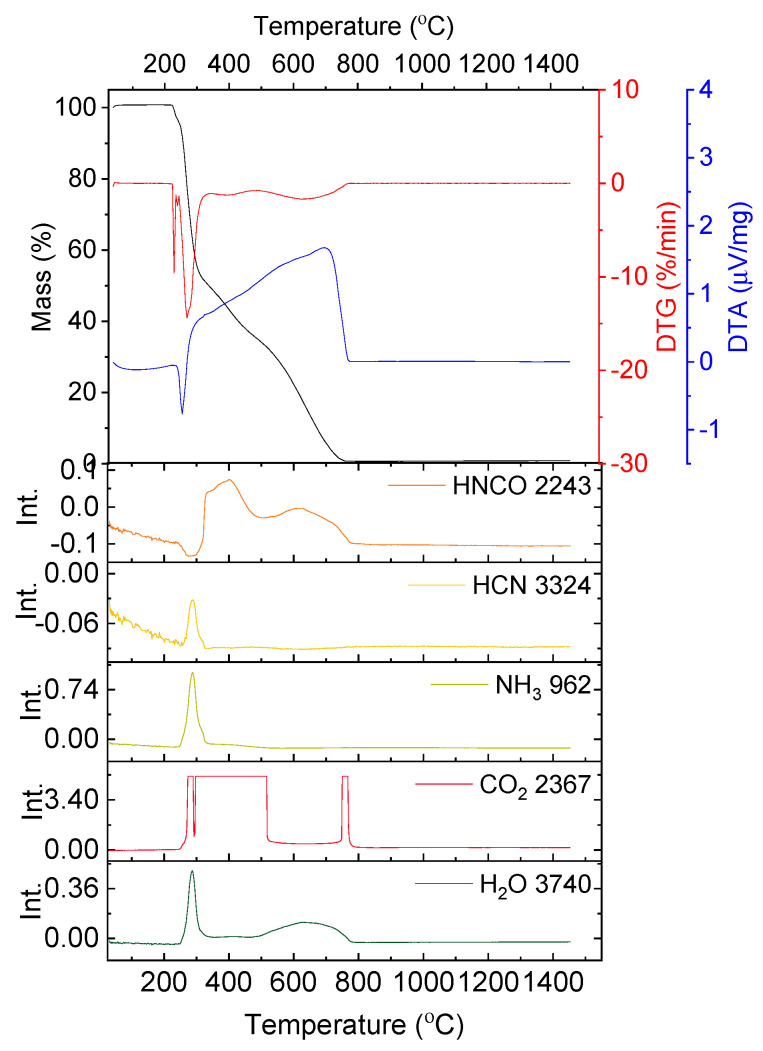
Thermal analysis results of the decomposition of glycine in synthetic air; absorbance intensities of traces for wavenumbers: 2243, 3324, 962, 2367 and 3740 cm^−1^ corresponding with absorbance peaks of HCNO, HCN, NH_3_, CO_2_ and H_2_O, respectively.

In Figure 2 the results of the thermal analysis for glycine are presented.

The decomposition of glycine begins at a temperature of 228.6 °C. Up to a temperature of 303.8 °C the mass loss amounts to 46.26% with two overlapping maximum mass loss rates at T = 235.0 and 269.7 °C and with a sharp endothermic peak at 252.3 °C. In the exhaust gases HCNO, HCN, NH_3_, CO_2_ and H_2_O were detected.

The next decomposition stage occurs at a temperature range of 269.7–473.6 °C with a minimum on DTG at T = 391.9 °C and no distinctive effect on the DTA curve. The mass loss equals 18.46% and is connected with the emission of CO_2_ and HNCO.

The last stage of decomposition is the residual burnout (exothermic peak at T = 695.2 °C) and it ends at 835.8 °C (Δm = 35.34%).

According to literature [24] the decomposition begins with the emission of NH_3_. Simultaneously, glycine can undergo condensation and cyclization reactions through dehydration reactions (Equations (3) and (4)). Subsequently, HCN, HNCO and CO is emitted due to selective cracking of cyclic amides. In air HNC and CO oxidize to HNCO and CO_2,_ respectively (5) [24].


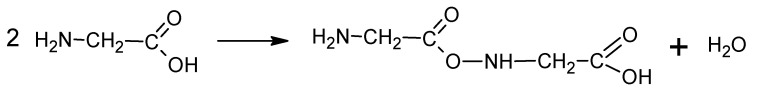
(3)


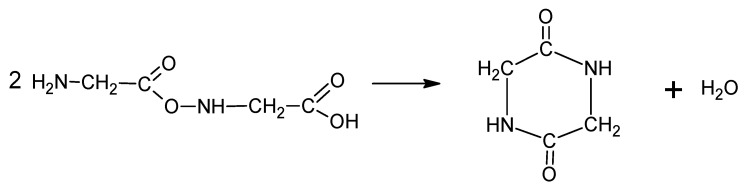
(4)


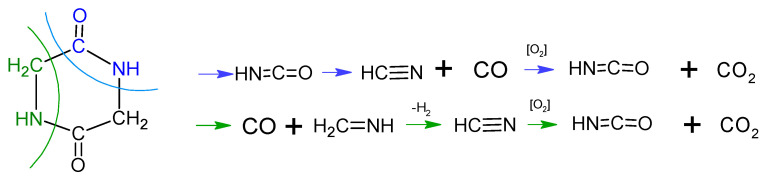
(5)

**Figure 3:**
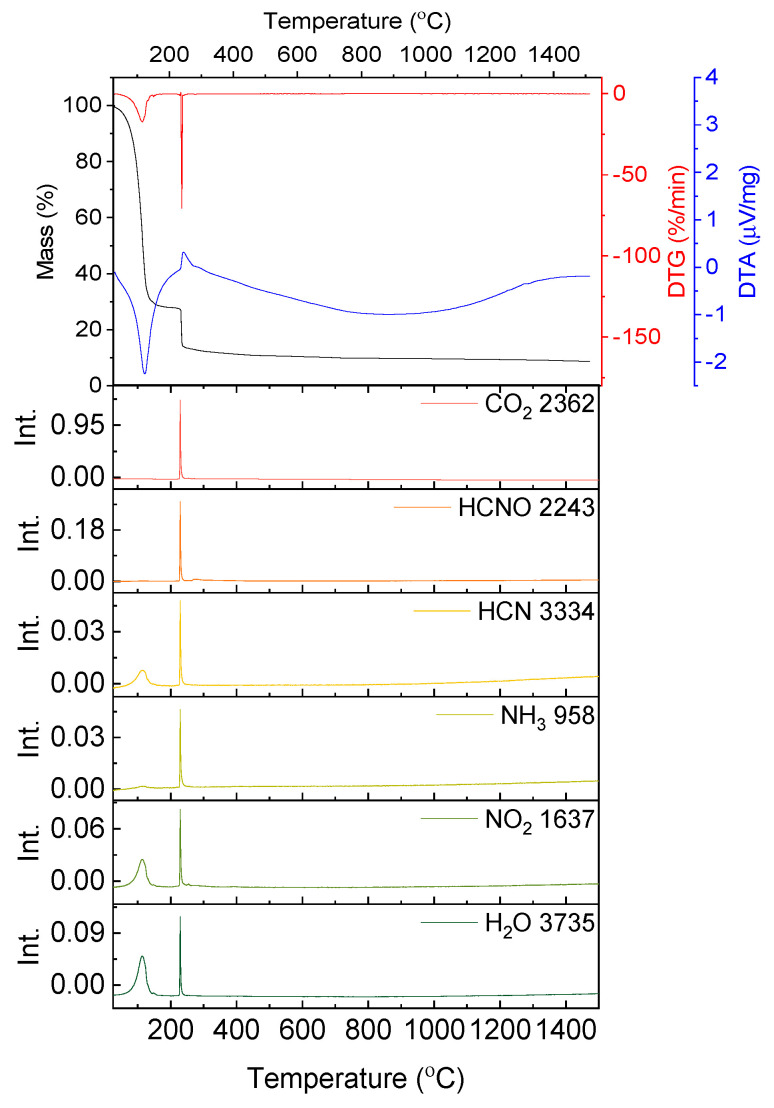
Results of the thermal analysis performed in synthetic air on the water solution containing yttrium nitrate and glycine; absorbance intensities of traces for wavenumbers: 2362, 2243, 3324, 958, 1637 and 3735 cm^−1^ corresponding to absorbance peaks of CO_2_, HCNO, HCN, NH_3_, NO_2_ and H_2_O, respectively.

In Figure 3 the results of the thermal analysis for the solution containing stoichiometric amounts of yttrium nitrate hexahydrate and glycine are presented. The measurement was conducted in synthetic air flow, to best imitate the conditions of the synthesis, which takes place in an open quartz beaker.

The slope on the TG curve produced during the thermogravimetric measurement of the solution containing yttrium nitrate and glycine begins below 100 °C. The first endothermic effect (with the peak at T = 124.3 °C) continues up to a temperature of 182.0 °C and is connected with a mass loss of 71.79%. The mass loss is attributed mainly to the evaporation of water. However, on the FT-IR spectra signals resulting from the presence of HCN, NH_3_ and NO_2_ are also visible (Figure 3). This is surprising, as these compounds result from the degradation of glycine and yttrium nitrate, which according to the results presented in Figure 1 and Figure 2 should be stable in this temperature range.

**Figure 4:**
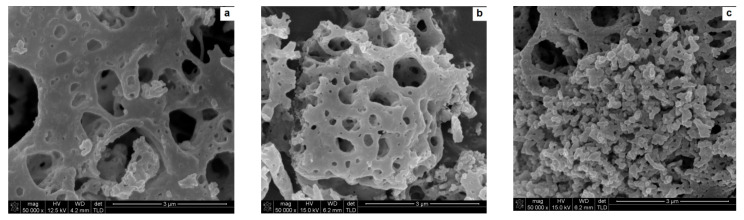
Micrographs of yttria nanopowder obtained by solution combustion synthesis (SCS) by the reaction of yttria nitrate with glycine: (**a**) not calcined, (**b**) calcined at a temperature of 800 °C, (**c**) calcined at a temperature of 1100 °C.

At a temperature of 238.2 °C the red-ox reaction between yttrium nitrate and glycine begins. It is distinguished by the exothermic peak on the DTA curve (T_peak_ = 244.1 °C) and an abrupt weight loss (Δm = 15.93%, Figure 3). At a temperature of 263.0 °C the process ends and with further temperature increase minor mass loss is observed (Δm = 3.96%). On FT-IR spectra not only CO_2_ and H_2_O, but also HCN, HCNO, NH_3_ and NO_2_ are detected. The yttria powders prepared by solution combustion synthesis using glycine and yttrium nitrate hexahydrate were investigated in terms of microstructure (Figure 4), particle size distribution and specific surface area (Table 1).

The synthesized powders are characterized by a highly porous microstructure (Figure 4a). The cumulants mean measured by technique of dynamic light scattering (DLS) is 2354 nm with a broad particle distribution (Pd = 1498 nm).

To burn out the substrates’ residues the powders were calcined. After calcination at a temperature of 800 °C the powders sponge-like microstructure remained intact (Figure 4b). The DLS analysis provides information about average particle size expressed in the cumulants mean of 2649 nm while d_V50_ = 0.9 µm (Table 1). These strong discrepancies result from high polydispersity of particle size (expressed in a high value of polydispersity width—Pd), also visible in the SEM image (Figure 4b) where a micrometric sized particle is accompanied by some smaller grains at the image boundary. The BET surface is 19.7 m^2^/g demonstrating a relatively high level of surface development.

Powders calcined at a temperature of 1100 °C reveal a finely grained microstructure. The microstructure transformation occurs without mass loss (Figure 3). The fine (about 100 nm in diameter) grains occur in agglomerates, with Z_ave_ = 1338 nm and d_V50_ = 1190 nm. Together with agglomerate size, BET surface is decreased as well and equals 11.5 m^2^/g (Table 1). During calcination at a temperature of 1100 °C the disordered matter in nanostructures of high surface energy undergoes diffusion and reorganization into grains. The “sponge-like” structure undergoes conversion—its thin walls disappear and in its place uniform globular grains are produced. The specific surface decreases in the process of matter diffusion and grain formation, but the globular grains are connected by van der Waals forces or sintering necks. Such structure is more probable to disintegrate than the initial “sponge-like” aggregates which is portrayed in the DLS analysis. This kind of structure is still not beneficial for ceramic technology, as agglomerates are very difficult to densify during conventional pressing techniques and may cause fluctuations in density in the bulk of the pressed sample.

**Figure 5:**
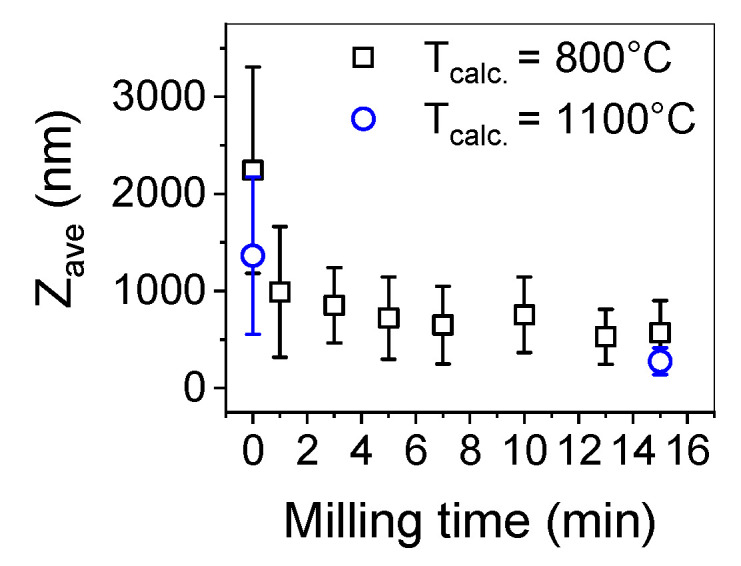
Particle diameter (expressed in cumulants mean—Z-ave) of yttria powder obtained by the SCS method.

**Figure 6:**
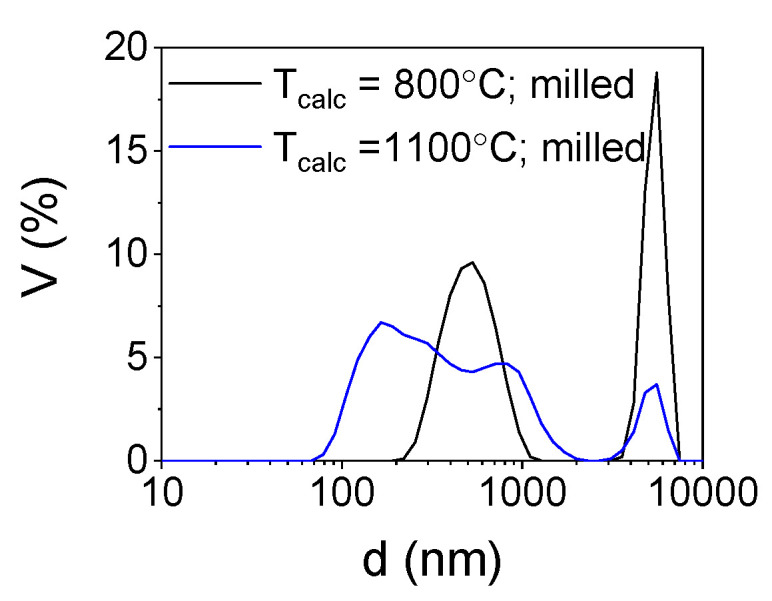
Particle size distribution of the milled powders.

To deagglomerate the obtained powder high-energy milling was implemented. In Figure 5 the trend of cumulants mean vs milling time and in Figure 6 size distributions of the milled powders are presented.

**Figure 7:**
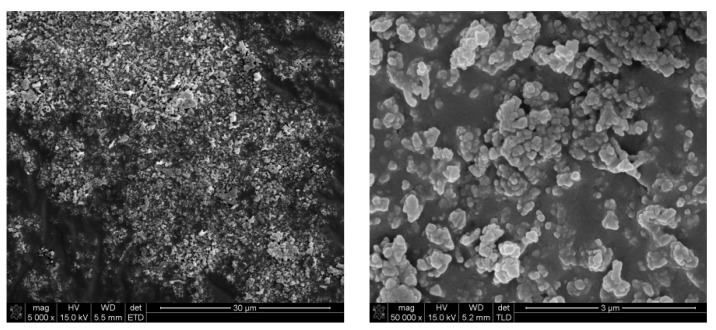
Micrographs of cryo-granulated yttria obtained by solution combustion synthesis through the reaction of yttria nitrate with glycine calcined at a temperature of 1100 °C after milling for 15 min in an attritor mill at a speed of 1000 rpm.

**Figure 8:**
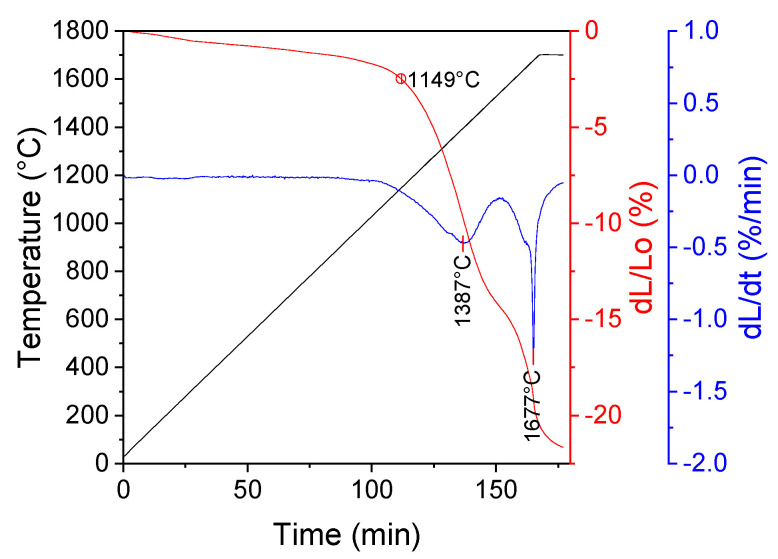
Dilatometric curve of a sample prepared from yttria powder obtained by method of the SCS with glycine.

In order to estimate the optimal time of milling the size distribution of yttria powder calcined at a temperature of 800 °C was measured after 1, 3, 5, 7, 10, 13 and 15 min (Figure 5). After 10 min, the particle size was about 500–600 nm and remained unchanged until maximum milling time which was 15 min. Basing on these observations and previous experience considering deagglomeration of yttria in water using an attritor mill [26], the milling time was set for 15 min and yttria powders were milled in these conditions. The results of particle size measurements of the milled powders are presented in Table 1 and in Figure 5. The size distribution of yttria powder calcined at the temperature of 800 °C revealed some agglomeration, as agglomerates or aggregates of 5 µm are visible. Agglomerates of powder calcined at a temperature of 1100 °C were disintegrated more effectively during milling (Z_ave_ = 275 nm and d_V50_ = 352 nm) which is consistent with the microstructural observations of the powder before milling. The agglomerates visible in Figure 4c underwent partial disintegration, the particle size distribution showed in Figure 6 indicates that the milled powder is trimodal with peaks at 0.275, 0.850 and 5 µm, which suggests that most of the agglomerates were disintegrated into particles with diameters of about 275 nm with some 3–4 particle agglomerates (d = 850 nm) and some bigger agglomerates left intact. In Figure 7 the SEM images of the milled powders are presented, where particles of about 100–150 nm with some bigger agglomerates are visible. This powder was cryo-granulated with the addition of binder and plasticizer, die pressed and densified in a cold isostatic press under a pressure of 150 MPa before taking dilatometric measurements (Figure 8). The suspension used for cryo-granulation was very diluted (c_solids_ ≈ 5 vol%), which is the reason why the powder does not appear in proper granules. Instead it occurs as separate particles and small agglomerates (Figure 6).

The sintering starts at a temperature of 1149 °C and proceeds in two steps: the first—with the maximum sintering rate at a temperature of 1387 °C (dL/L_0_ = 14.33%) and the second with the maximum sintering rate at T = 1677 °C (dL/L_0_ = 7.33%).

## 4. Discussion

The general formula for the red-ox reaction of solution combustion synthesis (Equation (1)) suggests that the byproducts consist of the non-toxic gases: CO_2_, H_2_O and N_2_. However, the FT-IR analysis of the gases emitted during the reaction showed also such specimens as HCN, HNCO, NH_3_ (Figure 3). This indicates that the SCS reaction (1) conducted even in a very well controlled environment with homogenous temperature distribution (small sample in crucible and TGA chamber) is accompanied by decomposition reactions of the substrates: yttrium nitrate and glycine.

What is more, it was observed that below the redox reaction ignition point (238 °C) the presence of HCN, NH_3_ and NO_2_ was detected on FT-IR spectra (Figure 3). The specimens occur already at a temperature of about 124 °C.

Such observations have been also made by Biamino et al. [27] in the investigation of SCS with urea as fuel. In the aforementioned work [27] it was suggested that the emission of nitrate oxides derives from the direct reaction of nitrate with urea, which occur at a temperature below the reaction ignition point. In case of the reaction of yttrium nitrite with glycine signals deriving from NO_2_ and HCN are visible in FT-IR spectra already at a temperature of about 124 °C (Figure 3). According to [27] the corresponding reaction of glycine and yttrium nitrite can be described as follows (6, 7):

26 Y(NO_3_)_3_ + 6 NH_2_CH_2_COOH → 13 Y_2_O_3_ + 84 NO_2_ +12 CO_2_ + 15 H_2_O (7)

2 Y(NO_3_)_3_ + 24 NH_2_CH_2_COOH → Y_2_O_3_ + 30 HCN + 18 CO + 45 H_2_O (8)

In both cases the reaction proceeds with the formation of carbon oxides which were not detected during the measurement below the ignition point (238 °C). Reactions 6 and 7 may take place during the exothermic reaction after reaching the ignition point, as HCN and NO_2_ were detected then, together with carbon dioxide (the measurement was carried out in air which can cause the oxidation of carbon monoxide and cyanic acid to isocyanic acid). This suggests that the reduction of the nitrate does not cause a complete degradation of the glycine carbon chain. Presumably, the presence of nitrate in the solution can trigger the first step of glycine degradation i.e., removal of ammonia, which is visible in FT-IR spectra and can cause a degradation of the cyclic amides (5). Ammonia and intermediate compounds derived from the decomposition of cyclic peptides may react with nitrite in accordance with the following general formulas (8, 9):

14 Y(NO_3_)_3_ + 6 NH_3_ → 7 Y_2_O_3_ + 48 NO_2_ + 9 H_2_O (9)

4 Y(NO_3_)_3_ + 6 H_2_CNH → 2 Y_2_O_3_ + 6 HCN + 12 NO_2_ + 6 H_2_O (10)

The assumption is consistent with macroscopic observations as precipitation was observed when the solutions containing the reagents were left to age for a month at room temperature. This suggests that a reaction between reagents took place.

Combustion synthesis is a widely used method for the production of nanopowders in both, laboratory and semi-technical scale. The presented results indicate that for a scaling-up of the process special precautions must be undertaken, which will provide for the neutralization of the hazardous nitrogen derived compounds.

The reaction of yttrium nitrate with glycine leads to the fabrication of nanostructured powder. The SEM observation of the powders showed an agglomerated, “sponge-like” microstructure of the particles. The structure remains stable at 800 °C as the microstructure of the powder remains intact after calcination at this temperature. Such morphology may be beneficial in some applications [1,2,3,4]. However, for ceramic technology the agglomeration is undesired as the densification of the nanopowder is severely hindered. After calcination at a temperature of 1100 °C the microstructure of the powder underwent modification and the “sponge-like” structure transformed into agglomerates consisting of globular grains with a diameter of about 100 nm. Milling of the powder calcined at a higher temperature was more effective as the particle diameter measured by method of DLS was decreased to 275 nm. Despite the milling, some agglomeration was observed in the size distribution curve (Figure 6). Further studies will be focused on optimization of milling conditions.

Sintering of calcined powder starts at a temperature of 1149 °C and proceeds in two distinctive stages. Such behavior indicates that the sintering process is divided into two stages: densification of spherical particles (reorganization of particles without particle growth) and grain growth, which was also observed by other researchers [28]. Another explanation of this phenomenon is that the first densification stage corresponds to the sintering of grains within the agglomerates, afterwards the sintering of the agglomerate domains and the separate particles takes place [29,30].

## 5. Conclusions

Solution combustion synthesis is an effective method for yttria nanopowder fabrication. The high-temperature self-propagating red-ox reaction between yttrium nitrate and glycine lowers the temperature of the nitrate decomposition and yttria formation from 642 to 263 °C. The vast amounts of gases emitted during decomposition implies the formation of nanoparticles. The yttria powders obtained by method of the SCS have a high sintering ability an can be applied in ceramic technology.

## Figures and Tables

**Table 1:** Particle size measured by method of dynamic light scattering (DLS) and calculated from BET specific surface area of yttria powders obtained by using the SCS method (Z_ave_—cumulants mean, Pd—polydispersity width, d_V50_—median diameter of the particle size distribution, S_BET_—specific surface area, d_BET_—BET equivalent spherical particle diameter).

	Calcination Temperature, °C	Z_ave_, nm	Pd Width nm	d_V50_, nm	S_BET_, m^2^/g	d_BET_, nm
not milled	not calcined	2354	1498	1500	-	-
	800	2694	2319	859	19.7	61
	1100	1338	813	1190	11.5	104
15 min of milling in an attritor mill	800	570	329	747	-	-
	1100	275	137	352	-	-
